# Pyruvate dehydrogenase complex integrates the metabolome and epigenome in memory T cell differentiation in vitro

**DOI:** 10.21203/rs.3.rs-2464392/v1

**Published:** 2023-01-27

**Authors:** Tatiana N. Tarasenko, Payal Banerjee, Julio Gomez-Rodriguez, Derek Gildea, Suiyuan Zhang, Tyra Wolfsberg, Lisa M. Jenkins, NISC Comparative Sequencing Program, Peter J. McGuire

**Affiliations:** NIH: National Institutes of Health

**Keywords:** T cells, pyruvate dehydrogenase, mitochondria, glycolysis, epigenetics

## Abstract

**Background:**

Modulation of metabolic flux through pyruvate dehydrogenase complex (PDC) plays an important role in T cell activation and differentiation. PDC sits at the transition between glycolysis and the tricarboxylic acid cycle and is a major producer of acetyl-CoA, marking it as a potential metabolic and epigenetic node.

**Methods:**

To understand the role of pyruvate dehydrogenase complex in T cell differentiation, we generated mice deficient in T cell pyruvate dehydrogenase E1A (*Pdha*) subunit using a CD4-cre recombinase-based strategy. To control for the contribution of exogenous metabolites in vivo, we conducted our T cell functional studies in vitro. T cells were differentiated into memory and effector T cells using standardized protocols. Cells were analyzed using stable isotopic tracing studies, metabolomics, RNAseq, ATACseq, ChIPseq and histone proteomics.

**Results:**

Herein, we show that genetic ablation of PDC activity in T cells (*TPdh*^*−/−*^) leads to marked perturbations in glycolysis, the tricarboxylic acid cycle, and OXPHOS. Due to depressed OXPHOS, *TPdh*^*−/−*^T cells became dependent upon substrate level phosphorylation via glycolysis. Due to the block of PDC activity, histone acetylation was reduced, as were most other types of post translational modifications. Transcriptional and functional profiling revealed abnormal CD8^+^ memory T cell differentiation in vitro.

**Conclusions:**

Collectively, our data indicate that PDC integrates the metabolome and epigenome in memory T cell differentiation. Targeting this metabolic and epigenetic node can have widespread ramifications on cellular function.

## Background

Pyruvate dehydrogenase complex (PDC) is a tripartite mitochondrial matrix enzyme which consists of pyruvate dehydrogenase (E1), dihydrolipoamide acetyltransferase (E2) and dihydrolipoamide dehydrogenase (E3). This enzyme complex is responsible for the oxidation of pyruvate to acetyl-CoA, the activated form of acetate, CO_2_ and NADH, and serves as the link between glycolysis and the tricarboxylic acid cycle (TCA). The major metabolic fates of acetyl-CoA include the provision of carbon skeletons for the TCA cycle, and the biosynthesis of fatty acids and cholesterol.

In addition to its contributions to cellular energy, acetyl-CoA may also serve as a substrate for the post translational modification of histones. During histone acetylation, an acetyl group from acetyl-CoA is transferred to the primary amine in the ε-position of the lysine side chain. This epigenetic modification results in neutralization of positive electrostatic charge, ultimately affecting DNA access and transcription. Since one of the major sources of acetyl-CoA is glycolysis, it is not surprising to find that histone acetylation is directly modulated by glycolytic flux and cellular metabotype^1^. Therefore, PDC may serve as a major node in T cells, integrating metabolism and epigenetics.

T cell activation and differentiation involve a series of coordinated steps involving metabolic, epigenetic and subsequently, transcriptional reprogramming ^2-4^. Regarding metabolic reprogramming, activated T cells awake from their quiescent state of OXPHOS dependence to develop a Warburg-like metabotype. Upon differentiation, this metabotype is retained (e.g., inflammatory T helper 1 cells) or recedes back to OXPHOS (e.g., memory T cells). Following these rapid metabolic changes, T cells experience changes in chromatin accessibility and transcription, indicating that these processes are temporally linked and dependent^5^. As such, T cell activation and differentiation serves as an excellent in vitro model for studying the intersection between metabolic perturbations and epigenetics.

Despite its central position in metabolism, the role of PDC in integrating the metabolome and epigenome in T cells remains unclear. We hypothesized that ablation of PDC activity would have widespread metabolic and epigenetic consequences and lead to aberrant gene expression, ultimately impacting T cell differentiation. To understand the role of PDC in metabolism and its effects on the epigenome, we developed a mouse model of T cell *Pdha1 (E1)* deficiency using a cre recombinase-based strategy. In the present study, we defined the effects of PDC deficiency on the metabolome and epigenome during memory T cell differentiation in vitro. We specifically chose this in vitro strategy to control for the contributions of extracellular metabolites to T cell activation and differentiation^6^.

## Materials And Methods

### Murine model of TPdh^−/−^

B6.129P2-Pdha1^tm1ptl^/J mice were crossed with CD4-Cre transgenic mice (B6.Cg-Tg(Cd4-cre)1Cwi/BfluJ). Both strains were acquired from The Jackson Laboratory. The resultant mice were referred to throughout the paper as TPdh^−/−^. Male and female mice, 8-12 weeks were used for experiments. Animals were euthanized in a carbon dioxide chamber followed by cervical dislocation. All animal care and procedures were approved and authorized by the Animal Care and Use Committee of the National Human Genome Research Institute (NHGRI Animal Safety Protocol G-11-3). All experiments were performed in accordance with relevant guidelines and regulations. The studies herein were conducted and reported in accordance with ARRIVE guidelines.

### Immunoblot studies

For analysis, approximately 20 mg of protein was loaded on 4-20% Tris-glycine polyacrylamide gels and run at 150V for 1.5 h. Transfer to polyvinylidene difluoride membrane was done using the Trans-Blot Turbo Transfer System (Biorad, Hercules, CA). The membranes were blocked 1h room temperature in proprietary buffer (LI-COR Biosciences, Lincoln, NE). The membranes were probed with PDH and phospho-PDH (Abcam, San Francisco, CA) and b-actin (Sigma-Aldrich, St. Louis, MO). After washing the membranes three times (10 min each) with TBS 0.1% Tween 20, incubation with IRDye secondary antibodies was performed (LI-COR Bioscience, Lincoln, NE). Image capture and analyses were accomplished using an Odyssey Imager (LI-COR Bioscience, Lincoln, NE).

### Real time PCR

Extracted RNA (Pure link RNA mini kit, Thermo Fisher Scientific) was reverse transcribed to cDNA iScript Kit (BioRad). Reactions were cycled and quantitated with an ABI 7500 Fast Real Time PCR System (Applied Biosystems).

### Metabolomics

Randomly selected mice were euthanized in a carbon dioxide chamber followed by cervical dislocation and spleens were extracted. Isolated splenic T cells were sent for metabolomic analyses by Clarus Analytics (SanDiego, CA).

### Stable Isotope studies

T cells were stimulated for 24 hours with immobilized anti-CD3 and anti-CD28. All labeling experiments were performed with 1 million cells/mL in RPMI. Glycose free or glutamine free media were replaced by their respective uniformly ^13^C-labeled analog (i.e. [U-^13^C]glucose or [U-^13^C]glutamine; Cambridge Isotope Laboratories). Cells were cultured for 24 hours and then pelleted, and lysed in cold 50% methanol. Analyses were performed at the CRI Metabolomics Core, UT Southwestern. Lysates underwent three freeze-thaw cycles, followed by centrifugation to remove debris. The supernatants were evaporated, methoximated and derivatized by tert-butyl dimethylsilylation. Derivatized material (1 mL) was injected onto an Agilent 6970 gas chromatograph equipped with a fused silica capillary GC column (30 m length, 0.25 mm diameter) and networked to either an Agilent 5973 or 5975 Mass Selective Detector. The measured distribution of mass isotopologues was corrected for natural abundance of ^13^C^7^.

### Extracellular flux analysis

Oxygen consumption rate (OCR) and extracellular acidification rate (ECAR) were determined using a Seahorse XF96 analyzer (Agilent). T cells activated for 24 hours with anti CD3 and anti CD28 were attached with Cell-Tak (Corning) at 0.2 million cells/well in Seahorse Base Medium Minimal DMEM supplemented with 12mM glucose, 2mM glutamine and 1mM sodium pyruvate. Mitochondrial parameters were monitored using the Mitostress kit (Seahorse Biosciences) according to the manufacturer’s standard protocol.

### Flow cytometry

Single-cell suspension of tissues were prepared. Anti- CD4, CD8, Ly6C, CD62L, CD25 antibodies were purchased from BD Biosciences or ebioscience. Labeled tetramers (NIH tetramer core facility) were used to identify viral specific T-cells. Data were acquired on CytoFLEX Flow Cytometer (Beckman Coulter) and analyzed using FlowJo software (Tree Star). Cells were loaded with 2mM CTV (ThermoFisher Scientific) and proliferation was estimated on day 3 by FACS. Cells were stimulated with biotinylated anti CD3 and crosslinked with streptavidin (Sigma-Aldrich). Flow cytometry gating strategy is depicted in Figure S4.

### Cell isolation and proliferation assays

Pan T cells, CD8^+^ T and CD4^+^ cells were enriched using isolation kits (Miltenyi Biotec). Purity of T-cells was >95% in all cases. T-cells were stimulated with plate-bound anti-CD3 (5 μg/ml) and anti-CD28 (0.5 μg/ml) in glucose media.

### In vitro differentiation

In vitro differentiation assays were performed as published previously ^8^. Briefly, naive T cells (CD4^+^, CD44^low^, CD62L^hi^, CD25^neg^) were purified by cell sorting. Purity was greater than 99%. Sorted T cells (2 × 10^5^) were co-cultured at a ratio of 1:5 with mitomycin-treated T-depleted splenocytes as APCs in 48-well plates in the presence of anti-CD3, anti-CD28 under differentiation conditions for 3 days. Th1 conditions: 40 ng/ml IL-12 and anti-IL-4. Th2 conditions: 20 ng/ml IL-4 and 10 μg/ml anti-IL-12. Th17 conditions: 20 ng/ml of IL-6, 5 ng/ml of TGF-β1, anti-IL-4, anti-IFN-γ, and anti-IL-12. Treg conditions: 100 U/ml hIL-2, 5 ng/ml TGF-β1, and 10 μg/ml of each anti–IL-4, anti–IFN-γ, and anti–IL-12 antibodies. Antibodies were purchased from BioXcell. OT-I cells were activated with OVA-peptide for 3 days. To differentiate into TE or TM, cells were cultured in the presence of IL-2 or IL-15 (10ng/mL) for 4 days, respectively ^9^. Sodium acetate (25mM) and LDH inhibitor GSK2837808A (10mM), (Tocris, Bristol, UK) were added to differentiation media.

### T cell killing assays

Splenocytes (10^6^/mL) from OT1 mice were stimulated in RPMI + 10% FCS with 1mM OVA peptide for 3 days, washed and cultured with IL2 as above for 3 more days. Targets EL-4 cells loaded with Cell Trace Violet (C34557) at 2uM in PBS for 10 minutes then quenched and washed. Cells were pulsed with 1uM SIINFEKL peptide for 30-60 minutes at 37 degrees. Target cells were incubated with activated OT-1 cells, at varying ratios (1:5, 1:10, 1:20 in 200 mL media for 4 hours. Stained w/APC-anti-CD8 and live/dead for 30 minutes on ice. Acquired on Beckman CytoFLEX cytometer.

### RNAseq

Poly-A selected RNA-seq libraries were constructed from 1 mg total RNA using the Illumina TruSeq RNA Sample Prep Kits, version 2. The resulting cDNA was fragmented using a Covaris E210. Library amplification was performed using 11 cycles to minimize the risk of over amplification. Unique barcode adapters were applied to each library. Libraries were quantitated by qPCR using the KAPA Library Quantification Kit (KAPA Biosystems) and pooled in an equimolar ratio. The pooled libraries were sequenced on a NovaSeq 6000 with version 1 chemistry. At least 90 million 150-base read pairs were generated for each individual library. Data was processed using RTA 3.4.4.

### ATACseq

Tagmented DNA samples were amplified to add single indexed adapters using the Kapa HiFi PCR MasterMix (Roche). The final libraries were twice purified using Ampure XP PCR Purification Beads (Agencourt). The libraries were pooled and then quantitated by qPCR. The pool balance was checked by performing a MiSeq run using a MiSeq Nano kit, version 2. The percentage of each library in the pool was determined from the demultiplexing and was used to rebalance the pool before sequencing.

The pooled libraries were sequenced on an SP flow cell on a NovaSeq 6000 using version 1.5 chemistry to achieve a minimum of 61 million 101 base read pairs. Raw sequence data were processed using RTA version 3.4.4. ATACseq reads were trimmed using Trimmomatic (v. 0.39) to ensure removal of adapter and transposase sequences. Adapter sequences compiled for Trimmomatic (Nextera-PE-PE.fa) were used for adapter trimming, with the option ‘ILLUMINACLIP:NexteraPE-PE.fa:2:30:10:8:true’. ATACseq reads were aligned to the mouse GRCm38/mm10 reference genome sequence (EMSEMBL) using BWA MEM (v. 0.7.17). ATACseq reads that mapped unambiguously to one genomic site were retained by filtering out reads tagged with ‘XA:Z:’ or ‘SA:Z:’ in the SAM file generated by BWA. ATACseq reads that mapped to blacklisted regions of GRCm38/mm10 (ENCFF547MET.bed; https://www.encodeproject.org/files/ENCFF547MET/), were removed due to difficulty in accurately mapping reads to these genomic regions. MAC2 software (v. 2.2.7.1) was used to identify regions of open chromatin from the ATACseq data, with options ‘-g mm’ to specify genome size and ‘-f BAMPE’ to specify paired-end mapping of ATACseq data.

### ChIPseq

Acetylated histones were immunorecipitated using anti-acetyllysine histone antibodies (ab1191, Abcam, Waltham, MA) for ChIPseq or ChIP PCR. For ChIPSeq, libraries were constructed from 50 ng of ChIP DNA using Ovation Ultralow System V2 1-96 with 15 cycles of PCR amplification. The final libraries were twice purified using Ampure XP PCR Purification Beads (Agencourt). The libraries were pooled and then quantitated by qPCR. The pool balance was checked by performing a MiSeq run using a MiSeq Nano kit, version 2. The percentage of each library in the pool was determined from the demultiplexing and was used to rebalance the pool before sequencing.

The pooled libraries were sequenced on a NovaSeq 6000 using version 1.5 chemistry to achieve a minimum of 37 million 51-base reads. The data were processed using RTA version 3.4.4.

First, quality control checks were performed using FastQC (0.11.9) (http://www.bioinformatics.babraham.ac.uk/projects/fastqc/) on raw sequence data in fastq format. The Phred scores for all samples were above 30, so we continued with the next steps. The fastq files were aligned to the mouse GRCm38/mm10 reference genome sequence (ENSEMBL) using BWA-mem (0.7.17). An important issue with ChIP-seq data concerns the inclusion of multiple mapped reads where the same reads are mapped to multiple loci on the reference genome. Including multiple mapped reads increase the number of usable reads and the sensitivity of peak detection; however, the number of false positives may also increase. Thus, we filtered the output BAM files using Samtools (1.15.1 ) view to retain only uniquely mapping reads. Blacklisted regions are largely comprised of sequences like major satellite repeats. These regions will show aberrantly high signal in all samples, thereby skewing normalization and often adding meaningless peaks. Thus, the reads overlapping with backlisted regions were removed from bam files using Samtools view. The locations of the blacklisted regions were downloaded from the ENCODE project (https://www.encodeproject.org/files/ENCFF547MET/). Next, we used MACS2 (2.2.6) to call broad peaks to identify areas in the genome that are enriched with aligned reads next to position of protein (histone) bound to DNA. Further, Diffbind (3.2, http://bioconductor.org/packages/release/bioc/html/DiffBind.html) was used to identify the differentially enriched peaks between wildtype and PDH-mutant samples. The peak profiles were annotated using ChIPseeker (1.28.3) in R (version 4.1.0). For visualization of peaks in UCSC Genome Browser, the bam files were first sorted and indexed using Samtools sort and Samtools index respectively and the bedGraph files were converted to bigwig files using UCSC bedGraphToBigWig tool.

### Histone proteomics

Posttranslational modifications of histones were performed using previously published methods ^10^. Briefly, core histones were extracted purified using the “Histone Purification Mini Kit” (#40026, Active Motif, Carlsbad, CA) according to the manufacturer’s instructions. Histones were acid-extracted, enriched on ion-exchange columns and desalted by perchloric acid precipitation. The purified histones were resuspended in HPLC-grade dH2O (1.0 mg/mL). Proteomic analyses was performed by the Mass Spectroscopy Section of NCI (Bethesda, MD).

### Statistical analyses

All experiments were repeated 3 or more times and summary or representative data were presented as appropriate. All measurements were taken from discrete samples. Statistical analyses were performed using Prism (Graphpad Software). Summary statistics were generated for all data. Two-sided unpaired Student’s t-test was used for comparing two groups where the populations followed a normal distribution, similar variance, and were sampled independently. P-value of < 0.05 was statistically significant. Means were represented by a single line with standard error of the mean for variation.

### Data availability

The datasets generated during and/or analyzed during the current study are available from the corresponding author on reasonable request.

## Results

### PDC deficiency in T cells: TPdh^−/−^ mouse

**Figure 1:**
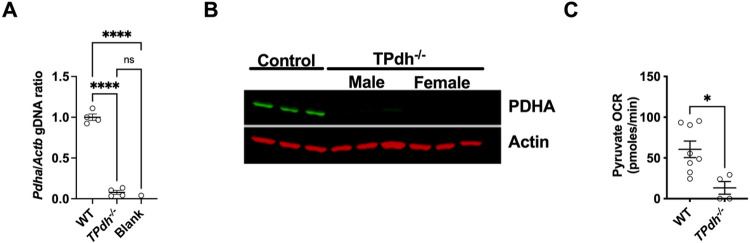
Mouse model of T cell pyruvate dehydrogenase complex deficiency. A) *Pdha* DNA in splenic T cells from *TPdh*^*−/−*^. CD4^+^ cre-recombinase was used to target T cells for deletion of *Pdha* locus. qPCR for *Pdha* was performed. N = 4 mice/condition. B) Immunoblot for PDHA from *TPdh*^*−/−*^ T cells. Total protein was extracted from splenic T cells. Immunoblots were probed for PDHA and normalized to actin (N = 3 / condition). C) Pyruvate oxidation in activated T cells. T cells were activated for 24 hours with CD3/CD28 and cultured in glucose free media supplemented with pyruvate as a carbon source. Extracellular flux analysis was performed. *** P < 0.001, **** P < 0.0001. Central line = mean, error bars = standard error of the mean.

To understand the effects of disruption of this critical metabolic node in T cells, we developed a model of T cell PDC deficiency by targeting Pdha using a CD4-cre recombinase. TPdh^−/−^ mice display normal litter sizes, body weight and length and life span (data not shown). To confirm deletion of the Pdha locus, we performed qPCR on gDNA from splenic T cells. TPdh^−/−^ T cells displayed an absence of Pdha (Figure 1A). Similar to humans, Pdha is encoded on the X-chromosome^11^. Therefore, to determine the efficacy of our cre-recombinase and the utility of male and female mice for experiments, we studied both sexes for the presence of PDHA by immunoblot (Figure 1B). PDHA was absent in both male and female mice, enabling us to use both sexes for subsequent experiments. Finally, we wanted to determine whether pyruvate could be oxidized by activated TPdh^−/−^ cells. To answer this question, splenic T cells from WT and TPdh^−/−^ mice were isolated and activated for 24 hours with CD3/CD28 stimulation. Extracellular flux analysis was performed where glucose was removed and replaced by pyruvate (Figure 1C). While WT cells readily oxidized pyruvate, TPdh^−/−^ cells were impaired, consistent with a block at the level of PDC.

### TPdh^−/−^ cells are dependent upon glycolysis

**Figure 2:**
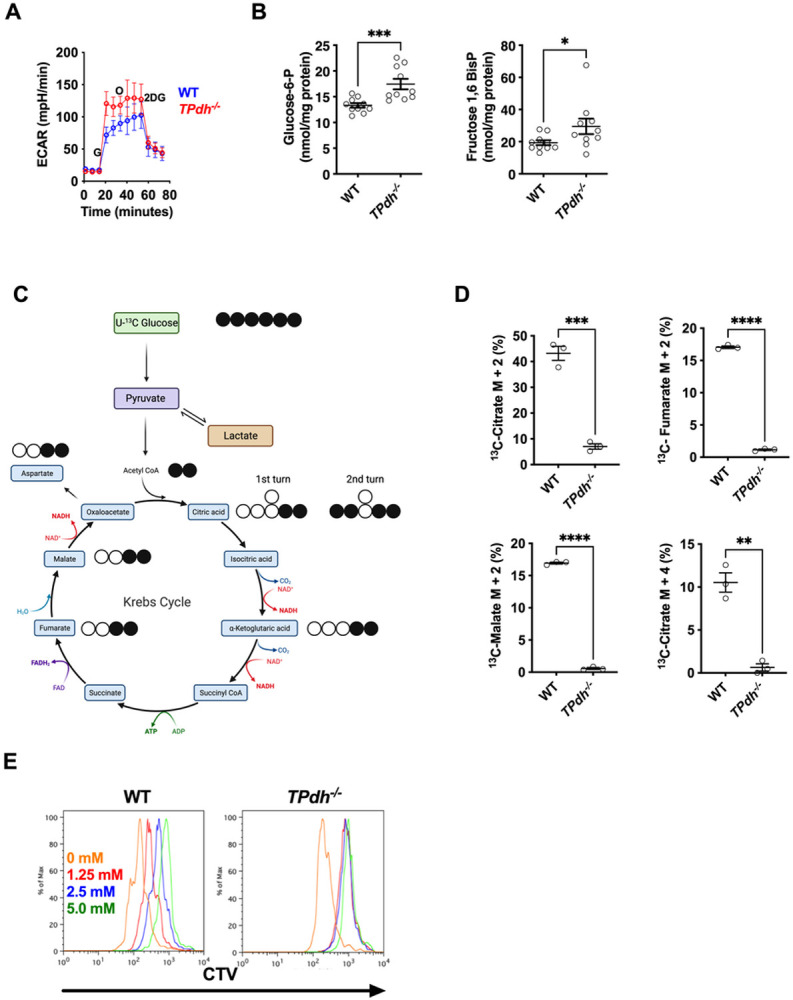
*TPdh*^*−/−*^ T cells display perturbations in glycolysis and disruption of tricarboxylic acid (TCA) cycle entry. A) Extracellular flux analysis of activated T cells. T cells were activated for 24 hours with CD3/CD28 antibodies. Glycolytic stress test was performed. B) Metabolomics for glycolytic intermediates. Splenic WT and *TPdh*^*−/−*^ T cells were stimulated for 24 hours as above. Cells were harvested and sent for metabolomic analysis. Metabolites were normalized to cellular protein levels. N = 10 mice/condition. C) Cartoon demonstrating use of uniform ^13^C-glucose as a carbon source for isotopomer labelling studies. D) Isotopologue labelling for citrate, malate, and fumarate. T cells were stimulated as above for 24 hours in the presence of ^13^C-glucose (N = 3 /condition). E) Glycolytic dependence in proliferating cells. Splenic T cells from WT and TPdh−/− T cells were stimulated as above and incubated with increasing concentrations of 2-deoxyglucose (2DG). Proliferation was measured by Cell Trace Violet (CTV) dilution via flow cytometry (N = 3 mice/condition). Representative of 3 or more experiments. Error bars = SEM. * P < 0.05, ** P < 0.01, *** P < 0.001, **** P < 0.0001. Central line = mean, error bars = standard error of the mean.

PDC is an important gatekeeper in metabolism, linking glycolysis and the TCA cycle. Since inhibition of PDC activity by PDK promotes aerobic glycolysis in culture and in vivo^12,13^, we predicted a similar increase in T cells with PDC deficiency. To profile glycolysis, we performed extracellular flux analysis following glucose injection on activated WT and TPdh^−/−^ splenic T cells (i.e., glycolytic stress test, Figure 2A). Following glucose injection, the extracellular acidification rate rose promptly with TPdh^−/−^ cells peaking about 50 mpH/min higher than WT. The addition of oligomycin to quantify glycolytic reserve resulted in a minimal increase in TPdh^−/−^ T cells, indicating that these cells were operating at their glycolytic maximum. To confirm increased utilization of glycolysis, we anticipated an accumulation of glycolytic intermediates. To identify these points of substrate accumulation, we conducted metabolomic analyses of glycolytic intermediates on activated T cells. While other glycolytic and pentose phosphate pathway intermediates were similar to WT (Figure S1A and S1B), TPdh^−/−^ T cells displayed elevated levels of glucose-6-phosphate and fructose 1,6 bisphosphate, the products of two key regulatory enzymes of glycolysis, hexokinase and phosphofructokinase, respectively (Figure 2B). The accumulations observed at critical metabolic checkpoints are consistent with enhanced glucose metabolism^14^.

The transition from glycolysis to the TCA cycle occurs in the mitochondria via PDC to produce acetyl-CoA, a critical metabolite for the TCA cycle ^15^. To confirm the interruption of glycolytic carbon transfer into the TCA cycle, we next examined the incorporation of ^13^C carbon from [U-^13^C] glucose into TCA cycle intermediates (Figure 2C). In WT, approximately 40% of the citrate pool was labelled as M + 2, indicating a considerable glucose-derived contribution to citrate through PDC (Figure 2D). Consistent with the genetic ablation of Pdha, the M+ 2 isotopologues of TCA cycle intermediates citrate, fumarate, and malate were essentially absent, with unlabeled (M + 0) intermediates comprising the predominant isotopologue (Figure 2D and S1C). The same was true for M + 2 aspartate, an amino acid derived from oxaloacetate. Cycling of the TCA from glucose derived carbon was also significantly depressed as reflected by the M + 4 citrate isotopologue (Figure 2D, lower right). To define functional glucose dependence of activated T cells, we studied proliferation by incubating stimulated T cells with increasing concentrations of 2-deoxyglucose (Figure 2E). While WT displayed a dose dependent effect, TPdh^−/−^ was found to have significant inhibition of proliferation at all doses of 2DG. Overall, our results not only confirm PDC deficiency, but also define a functional dependence of glycolysis on TPdh^−/−^ cells.

### Reprogramming of mitochondrial metabolism in TPdh^−/−^

**Figure 3:**
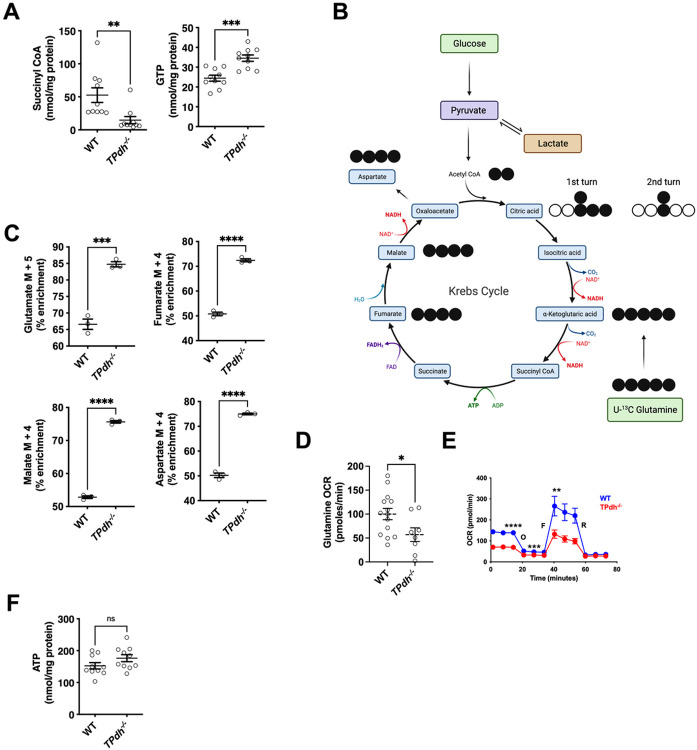
*TPdh*^*−/−*^ T cells maintain total cellular ATP despite perturbations in multiple energetic pathways. A) Metabolomics analyses. Splenic WT and *TPdh*^*−/−*^ T cells were activated as above for 24 hours. Cell pellets were collected and sent for metabolomic analysis. Metabolites were normalized to protein levels. N = 10 mice/condition. B) Cartoon demonstrating use of uniform ^13^C-glutamine as a carbon source for isotopomer labelling studies. C) Labelling of TCA cycle intermediates by ^13^C-glutamine in T cells activated for 24 hours (N = 3 mice / condition). D) Glutamine oxidation by extracellular flux analysis. Extracellular flux analysis was performed following the introduction of glutamine as the carbon source. (N = 14 WT mice, N = 8 *TPdh*^*−/−*^ mice. E) Extracellular flux analysis in T cells. T cells were activated for 24 hours using anti-CD3/CD28. Mitostress test was performed. N = 5-6 mice / condition. F) Total cellular ATP determined by metabolomics. WT and *TPdh*^*−/−*^ T cells were activated for 24 hours as above. Cell pellets were collected and sent for metabolomic analysis. Metabolites were normalized to protein levels. N = 10 mice / condition. Error bars = SEM. * P < 0.05, *** P < 0.001, **** P < 0.0001. Central line = mean, error bars = standard error of the mean.

The TCA cycle generates intermediates for sugars, amino acids, nucleic acids, and lipids, and provides reducing equivalents for oxidative phosphorylation^16-18^. Since the commitment of glucose derived carbon to the TCA cycle and aerobic metabolism was disrupted, we hypothesized that this would lead to metabolic adaptations^19,20^. To define these metabolic adaptations, we first profiled TCA cycle intermediates via metabolomics in CD3/CD28 activated T cells. Most TCA cycle intermediates were similar between TPdh^−/−^ and WT, suggesting a potential role for anaplerosis (Figure S2A and S2B). Indeed, multiple anaplerotic amino acids were depressed in TPdh^−/−^, with phenylalanine, tyrosine, isoleucine and valine being the most significantly affected (Figure S2C). Notably, one TCA cycle intermediate was markedly reduced in our metabolomics study. Succinyl CoA, the product of a-ketoglutarate dehydrogenase and substrate for succinyl CoA synthetase^21^, was decreased along with a significant increase in GTP (Figure 3A). These findings suggest generation of GTP by substrate level phosphorylation via succinyl CoA synthetase^22,23^. GTP can subsequently be converted to ATP by nucleoside-diphosphate kinase (NDPK)^24^, thus contributing to the overall cellular ATP pool. To profile the incorporation of alternate carbon sources into the TCA cycle in TPdh^−/−^, we employed [U-^13^C] glutamine (Figure 3B). Glutamine is converted to glutamate and subsequently a-ketoglutarate, the substrate that generates succinyl-CoA and downstream metabolites succinate, fumarate and malate. The M + 5 isotopomer of glutamate was increased in TPdh^−/−^ consistent with increased incorporation of ^13^C carbon (Figure 3C and S2D). Monitoring M + 4 isotopologues downstream showed enrichment of glutamine carbon in fumarate, malate, and aspartate (Figure 3C, Figure S2D).

Anaplerosis not only helps regulate rates of biosynthesis by augmenting substrate availability, but may also contribute to cellular energy status^21^. To test whether increased incorporation of glutamine carbon translated to enhanced OXPHOS in TPdh^−/−^, we next performed extracellular flux analysis on T cells activated as above. Increased incorporation into the TCA by glutamine did not result in enhanced OXPHOS, but rather a depression (Figure 3D), suggesting that this amino acid did not contribute to cellular energy status via OXPHOS. Based on our observations, we next sought to define OXPHOS by extracellular flux analysis. In activated TPdh^−/−^ T cells, basal respiration, ATP synthesis, maximal respiration and spare respiratory capacity were all depressed (Figure 3E). Based on our stable isotope and extracellular flux analyses, we suggest that in activated T cells, a portion of glucose is completely oxidized in the mitochondria. In addition, this oxidation of glucose may help set the pace for OXPHOS. In PDC, a depression in OCR was also seen when the long chain fat palmitate was used as a substrate, indicating that mitochondrial fatty acid oxidation was also reduced (Figure S2E). Despite significant depressions in FAO and OXPHOS in TPdh^−/−^ T cells, total cellular ATP was similar to WT, suggesting that substrate level phosphorylation was sufficient to account for the deficit (Figure 3F).

### Deficiencies in T cell differentiation in TPdh^−/−^ in vitro

**Figure 4:**
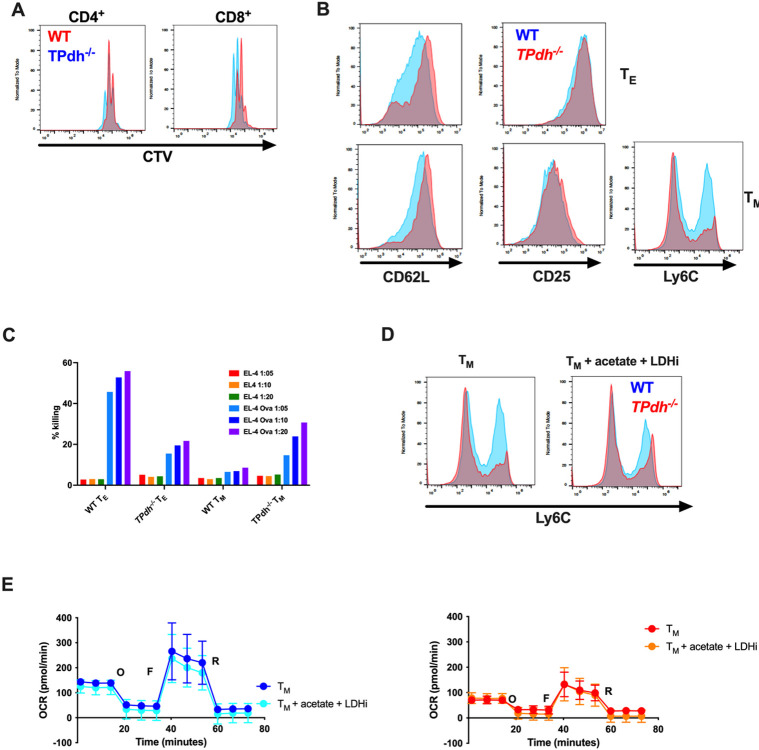
Abnormal T_M_ differentiation in *TPdh*^*−/−*^ cells. A) CD4^+^ and CD8^+^ T cell proliferation. Splenic T cells were isolated and stimulated with anti-CD3/CD28 for 72 hours. Proliferation was measured by Cell Trace Violet (CTV) dilution via flow cytometry. WT (blue), *TPdh*^*−/−*^ (red). B) *TPdh*^*−/−*^ T_E_ and T_M_ cell surface markers of differentiation. Following IL-2 or Il-15 treatment, cells were analyzed by flow cytometry for their appropriate surface markers. C) *TPdh*^*−/−*^ T cell killing assay. OT-I T_E_ and T_M_ cells were assessed for their ability kill EL-4 cell targets loaded with OVA peptide. D) Treated *TPdh*^*−/−*^ TM cells. In addition to IL-15 treatment, T cells were treated with acetate (10 mM) and a lactate dehydrogenase inhibitor (LDHi, 25 mM). Ly6C was determined by flow cytometry. E) Extracellular flux analysis of TM cells supplemented following treatment as in D). N = 5-6 mice/condition. Error bars = SEM. Flow cytometry and cell killing graphs are representative of multiple experiments. Experiments were repeated 3 or more times.

T cells play multiple roles in the adaptive immune system. In addition to killing infected host cells, T cells activate and coordinate multiple arms of the innate and adaptive immune response to pathogens, allergens and tumors^25^. To study T cell expansion prior to differentiation, we stimulated cells for 72 hours and measured proliferation by cell trace violet (CTV) dilution (Figure 4A). TPdh^−/−^ T cells displayed slightly compromised proliferation, with CD8^+^ cells showing a greater lag, consistent with their need for a more robust metabotype.

CD8^+^ T cells play a critical role in immunity, particularly viral infections. Following stimulation, CD8^+^ T cells rapidly proliferate and can differentiate into Ag-specific effector T (T_E_) cells or long-lived memory T (T_M_) cells that help protect against re-infection^26^. These differentiation states are dependent upon IL-2 and IL-15 stimulation, respectively^9^. To determine the impact of PDC deficiency on these cell types, we performed in vitro differentiation of T_E_ (IL-2) and T_M_ (IL-15) T cells (Figure S3A). TPdh^−/−^ T_E_ cells showed a retention of CD62L, a marker of T_M_ (Figure 4B). Conversely, TPdh^−/−^ T_M_ cells showed depressed expression of Ly6C, indicating a breakdown in memory differentiation. In adaptive immunity to viruses, CD8^+^ T_M_ cells comprise the memory pool, while T_E_ cells control viral proliferation by killing infected cells. To define the T_E_ phenotype, we performed cell killing assays using EL-4 cells. Interestingly, while TPdh^−/−^ T_E_ displayed reduced killing activity, T_M_ also displayed killing activity (Figure 4C), likely due to the retention of granzyme B activity (Figure S3B), suggesting abnormal differentiation.

We next asked whether metabolites provided by the extracellular environment could overcome the effects of PDC deficiency in T_M_ differentiation. Acetate, a ketone body produced during infectious states, has been shown to be involved in the acetylation of metabolic enzymes (e.g., GAPDH) and histones^6,27^. To test whether replacement of acetyl-CoA by acetate supplementation (10 mM) could aid in the differentiation of TPdh^−/−^ T_M_ cells, we performed in vitro differentiation with IL-15 as above. Since acetate alone did not produce changes in Ly6C expression (data not shown), we also employed a lactate dehydrogenase inhibitor (LDHi, 25 mM) to suppress Warburg metabolism and aid in the adoption of a T_M_ metabotype. With the addition of acetate and LDHi, we saw a slight improvement in Ly6C, suggesting skewing towards the T_M_ phenotype (Figure 4D). Interestingly, this improvement in T_M_ skewing in TPdh^−/−^ was not due to changes in the spare respiratory capacity of OXPHOS (Figure 4E), suggesting that the acetyl-CoA derived from acetate was involved in mechanisms of differentiation outside of bioenergetics.

### TPdh^−/−^ T_M_ display altered epigenetic signatures

**Figure 5:**
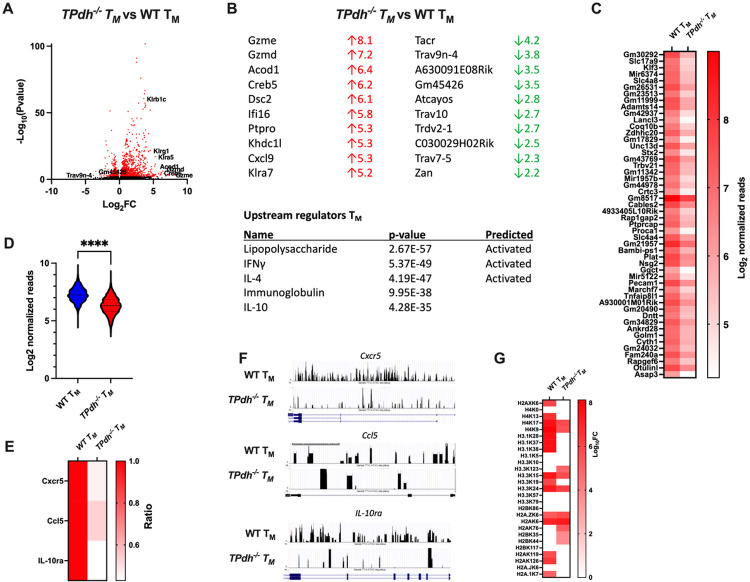
Genomic studies of T_M_ cells. Splenic T cells were differentiated into T_M_ cells using established protocols. RNA was extracted and submitted for RNAseq (N = 5 mice/condition). A) Volcano plot demonstrating differentially expressed genes. B) Gene ontology (GO) for differentially expressed genes. Gene ratio is the percentage of total differentially expressed genes in the given GO term. C) ChIPseq for T_M_ cells. Splenic T cells were differentiated as indicated above. Acetylated histones were immunopreciptated and DNA was sent for sequencing (N = 4 / condition). Top 50 genes detected by ChIPseq. D) Log_2_ normalized reads for ChIPseq. E) ChIP PCR of select targets identified by ChIPseq. Acetylated histones were precipitate as above. Genes involved in T_M_ differentiation which were also detected in ChIPseq were amplified using ChIP PCR. F) ATACseq of T_M_ cells. Splenic T cells were differentiated as above. Genes involved in T_M_ differentiation and identified in ChIPseq were examined for open regions of chromatin. Black peaks represent open regions of chromatin (N = 4/ condition). G) Histone proteomics. Histones were isolated from T_M_ cells and subjected to proteomic analysis for post-translational modifications. **** P < 0.0001.

Based upon our T_M_ studies, we hypothesized that TPdh^−/−^ T_M_ would display perturbations in gene expression due to aberrant epigenetic signatures, specifically, histone modification. Global histone acetylation levels are determined by glycolytic flux and PDC deficiency represents a major impediment to acetyl-CoA production^28^. To test this hypothesis, we began by characterizing expression signatures by RNAseq (Figure 5A). Compared to WT, TPdh^−/−^ T_M_ cells were found to have 16 downregulated genes and 414 upregulated genes (Log_2_ fold change >2, −Log_10_ P value > 1.3). Consistent with our functional assays, TPdh^−/−^ T_M_ cells displayed a divergent phenotype consistent with an inflammatory effector T cell phenotype. The top upregulated genes for TPdh^−/−^ T_M_ cells also included numerous granzymes and members of the killer-like receptor family, markers consistent with a T_E_ phenotype (Figure 5A and 5B, top) ^29,30^. Similarly, upstream regulators lipopolysaccharide and interferon gamma were also consistent with an inflammatory effector T cell phenotype. We interpreted this gene expression profile as abnormal differentiation of TPdh^−/−^ T_M_, with the cells retaining effector functions, an assertion which was supported by our cell killing assays (Figure 4D).

Since acetyl-CoA production is impacted by PDC deficiency, and is an essential component of gene regulation by histone modification, we next performed chromatin immunoprecipitation and sequencing (ChIPseq) studies by targeting acetylated histones. To visualize our genomic data, we constructed an MA plot (Figure S4A). In the figure, we found that WT T_M_ have nearly a log fold difference (purple) in bound sites when compared to TPdh^−/−^ T_M_. This translated into a generally lower number of genomic reads for the top 50 genes and at all loci in general (Figures 5C and 5D) for TPdh^−/−^. Although the number of acetylated sites was lower in TPdh^−/−^, the overall distribution of acetylated sites was similar between both groups (Figure S4B). To confirm our findings, we used ChIP PCR to probe several targets important for T_M_ differentiation that were identified by our ChIPseq (Figure 5E). Consistent with our ChIPseq results, TPdh^−/−^ T_M_ displayed a decreased ratio of acetylated target genes by ChIP PCR. These results were also consistent with our ATACseq results that showed limitations in chromatin accessibility for the aforementioned genes (Figure 5F). Based on these findings, we hypothesized that histone acetylation would be altered. To answer this question, we conducted a proteomic analysis for histones lysine acetylation. In general, TPdh^−/−^ T_M_ displayed decreased amounts of histone acetylation (Figure 5G). Overall, our results indicate perturbations in the epigenetic signature of TPdh^−/−^ T_M_, which impacts gene expression and by extension differentiation.

### PDC deficiency alters the broader epigenetic landscape of histone modifications

**Figure 6:**
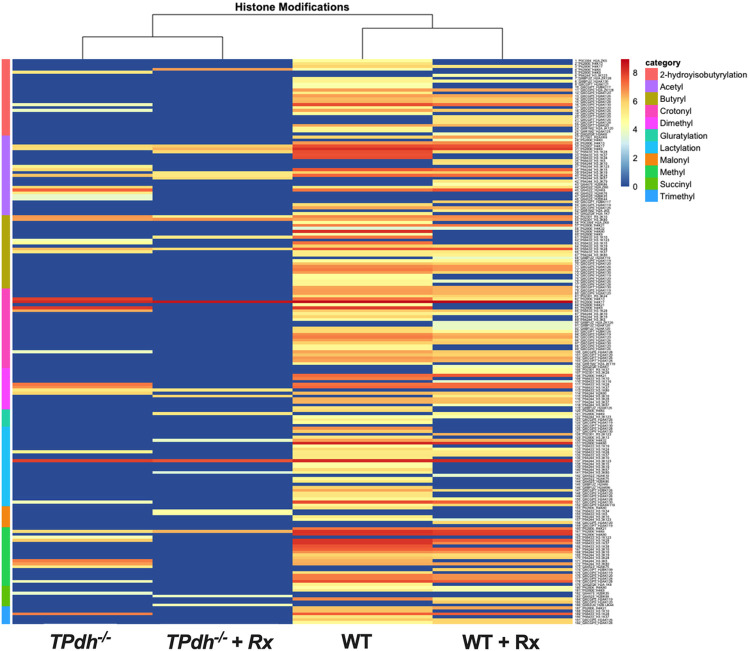
Altered histone modifications in TPdh^−/−^. T_M_ T cells were treated with acetate + LDHi and histones were isolated for proteomics (N = 4 / condition). Heatmap of histone modifications for untreated and treated T_M_ cells was constructed. Key indicates groups. Rx = treatment with acetate and LDHi. Each row indicates a histone site.

Metabolic rewiring and epigenetic remodeling via histone modification are interconnected and reciprocally regulate each other, thereby impacting cellular phenotypes. In addition to acetylation, cellular metabolism also contributes to other histone modifications including lactylation, butyrylation, crotonylation, glutarylation, malonylation, succinylation, methylation, demethylation, and trimethylation; many of which play a role in modifying gene expression profiles ^10,31,32^. Based on the metabolic rewiring seen in TPdh^−/−^ cells, we hypothesized that PDC deficiency would lead to broader perturbations in the histone code, beyond acetylation. To answer this question, we employed a histone proteomics approach. In general, TPdh^−/−^ memory T cells were not only deficient in acetylation, as expected, but were also deficient in nearly every type of histone modification studied. The overall fold change, and histone subtypes (Figure 6), were markedly different from WT. To investigate the response to cellular and environmental changes, we employed our acetate and LDHi treatment (Rx) as earlier. In general, there was a decrease in histone modifications seen in both WT and TPdh^−/−^ (Figures 6). In WT, the acetylation profiles remained mostly similar, with some replacements (e.g., acetylation for hydroxybutyrylation and lactylation at H2BK117 and HBK86, respectively). Following treatment, TPdh^−/−^ showed attrition of multiple histone modifications, especially on histones H2A and H2B, including a loss of acetylated sites. Overall, these results indicate that PDC deficiency leads to an alteration of the histone code during differentiation in response to intracellular and extracellular sources, explaining the incongruent phenotype seen in TPdh^−/−^ T_M_.

## Discussion

Metabolites derived from intermediary metabolism play an important role in epigenetics and can mediate important health outcomes such as immunity. T cells undergo metabolic reprogramming following activation to develop a metabotype that is not only conducive to the bioenergetic and substrate needs of the cell, but also contributes to the epigenetic landscape. Herein, we studied the metabolic and epigenetic effects of disruption of PDC in T cells in vitro. PDC deficiency leads to widespread perturbations in glycolysis, mitochondrial metabolism, and the epigenome. The result is defects in T cell differentiation and changes in the response to extracellular metabolites. Our results indicate that glycolysis is a significant contributor to histone acetylation, and PDC serves as an important metabolic and epigenetic node in T cell differentiation.

Following engagement of the T cell receptor, pyruvate dehydrogenase kinase 1 (PDK1) becomes activated in T cells, leading to the phosphorylation and subsequent inhibition of PDC ^33,34^. As a result, a smaller fraction of pyruvate (~ 40% by our stable isotope studies) is metabolized in the mitochondria, and T cells adopt a glycolytic metabotype. In our current model, *TPdh*^*−/−*^ T cells lack a critical component of PDC, resulting in a deficiency of this enzyme complex. As a result of this block, pyruvate is not fully oxidized and subsequently, OXPHOS is downregulated (by ~48%). In response, *TPdh*^*−/−*^ undergo metabolic rewiring and upregulate glycolysis as evidenced by our extracellular flux and metabolomic studies. Consequently, total cellular ATP levels are maintained via substrate level phosphorylation. This upregulation of aerobic glycolysis is dependent upon the regeneration of NAD^+^, a process which occurs in the cytoplasm via the conversion of pyruvate to lactate via lactate dehydrogenase (LDH)^35^. Indeed, our extracellular flux analyses support increased activity of LDH. Not only does this lead to an upregulation of glycolysis, but also a metabotype where glycolysis is operating at its maximum, unable to be pushed further. As a result, *TPdh*^*−/−*^ T cells become functionally dependent upon glycolysis, as indicated by our proliferation studies with 2DG.

In aerobic organisms, the TCA cycle is a sequence of chemical reactions used to produce energy through the oxidation of acetyl-CoA derived from glycolysis, fatty acid oxidation or amino acid metabolism^36^. In our *TPdh*^*−/−*^ T cell model, the TCA cycle undergoes metabolic rewiring involving anaplerosis due to a deficiency of acetyl-CoA from glycolysis. As a mechanism to maintain homeostasis, anaplerosis becomes essential in this case of loss of glycolytic carbon sources for the TCA cycle ^37^. One important source of anaplerosis is amino acids. In our study, *TPdh*^*−/−*^ T cells showed a depletion of multiple ketogenic amino acids (isoleucine, phenylalanine, tyrosine), as well as increased incorporation of glutamine into the TCA cycle as measured by stable isotopes. However, the replenishing of TCA cycle intermediates did not result in enhanced OXPHOS, indicating that its function lies beyond bioenergetics. One such important function may be the synthesis of aspartate from oxaloacetate seen in *TPdh*^*−/−*^. Aspartate synthesis in the setting of OXPHOS deficiency becomes an important pathway for producing DNA, RNA and protein in proliferating cells^38^. Furthermore, anaplerosis may also be enhanced by OXPHOS deficiency, leading to excessive anaplerosis^39^.

Since metabolism is intricately tied to T cell differentiation, it was not surprising to find abnormalities in *TPdh*^*−/−*^ CD8^+^ T cells. CD8^+^ T cells are highly energetic and have a requirement for intact OXPHOS. Unlike CD4^+^ T cells, activation of CD8^+^ T cells does not result in a complete shift to aerobic glycolysis^40^. In fact, OXPHOS levels increase and are an important source of ATP needed for cell proliferation. Therefore, impaired OXPHOS and enhanced glycolysis seen in PDC deficiency are more consistent with T_E_ cells and may partially account for this distinct phenotype seen in *TPdh*^*−/−*^ T_M_.

Beyond metabolic reprogramming, CD8^+^ T cell differentiation also involves epigenetic and subsequently, transcriptional reprogramming ^2-4^ PDC deficiency leads to a deficiency of acetyl-CoA, an important substrate for histone modification^31^. The importance of acetyl-CoA derived from glycolysis in differentiation has also been reported in a number of cellular systems. For example, glycolysis-mediated changes in acetyl-CoA and histone acetylation control differentiation in embryonic stem cells^31^. In *TPdh*^*−/−*^ T_M_ cells, histone acetylation was markedly depressed as shown in our proteomic and ChIP studies, suggesting that glycolysis is a significant source of acetyl-CoA in these cells. Therefore, the deficits seen in *TPdh*^*−/−*^ differentiation are mediated by metabolic and epigenetic perturbations.

In addition to acetylation, *TPdh*^*−/−*^ cells also showed perturbations in other histone signatures derived from other short chain CoA’s. The significance of these newer epigenetic signatures, are still be investigated, however, the general consensus is that these modifications at select residues alter gene expression ^10,31,32^. For example, crotonylation, derived from amino acid catabolism, shares many similar characteristics to acetylation. First, the substrate for crotonylation is a CoA ester. Second, crotonylation also occurs at the ε-amino group of Lys and alters histone charge. A key question that remains regarding lysine crotonylation, as with other acylations, is whether they are redundant, or have a distinct role from histone acetylation^32^. As the source of these short chain acyl CoA’s is intermediary metabolism, it is not surprising that this aspect of the histone code was altered in *TPdh*^*−/−*^. Besides glycolysis, TCA cycle and OXPHOS, changes in amino acid utilization was also altered, many of which are precursors for short chain acyl CoA’s.

In summary, our data demonstrate that PDC deficiency leads to metabolic and epigenetic perturbations, affecting CD8^+^ T cell differentiation in mice. Based on our findings, we propose that PDC occupies a major node in T cell intermediary metabolism by mediating both biochemical and epigenetic responses in activation and differentiation.
